# Spatial Aggregation and Biometric Variability of the Grass *Calamagrostis epigejos* (L.) Roth during Different Expansion Stages in Mesic Mountain Meadows

**DOI:** 10.3390/ijerph19137903

**Published:** 2022-06-28

**Authors:** Daniel Pruchniewicz, Ludwik Żołnierz

**Affiliations:** Department of Botany and Plant Ecology, Wrocław University of Environmental and Life Sciences, pl. Grunwaldzki 24a, PL-50-363 Wrocław, Poland; ludwik.zolnierz@upwr.edu.pl

**Keywords:** wood small-reed, degradation of vegetation, species diversity, self-thinning, growth strategy

## Abstract

*Calamagrostis epigejos* (L.) Roth is one of the most expansive clonal grass species. Despite many publications about its biology, the expansive mechanism of *C. epigejos* is relatively unknown. Therefore, the aim of this study was to determine: I. the dependency between *Calamagrostis epigejos* ramet density, habitat properties, and the biometric variability of the species; II. the relation between clone architecture and the diversity parameters and the productivity of grassland biocoenoses; III. the expansion strategy of *Calamagrostis epigejos* within mesic meadows, representing three stages of degradation. The research was conducted in the Central Sudetes (SW Poland). Ten transects were selected for the study, each representing *Arrhenatheretalia*-type meadows with patches degraded as a result of *Calamagrostis epigejos* expansion:initial, intermediate, and advanced. The phalanx strategy was observed within the studied range of the *Calamagrostis epigejos* expansion in the mesic mountain meadows. The study showed no relation between the *Calamagrostis epigejos* expansion and the phenomenon of ramet self-thinning, though it noted the influence of the habitat on the variability of its biometric features.

## 1. Introduction

Clonal plants play a particularly important role in creating the grassland-plant community. They can degrade those communities as well, when their success in competition with other species leads to strong domination.

Competition between plant species is an ecological process influencing development and driving species’ composition and the diversity of plant communities. Plant species competing for resources may develop various adaptation mechanisms. Berger et al. [1] indicate three main aspects of this issue: (1) density effects in which the mean size of plant individuals is related to density, (2) the emergence of size hierarchies within a population or community, and (3) ‘self-thinning’, i.e., density-dependent mortality of individuals. All these mechanisms may be influenced by a species dynamically expanding within a grassland community. 

The success of clonal plants in conquering new areas within a community can be attributed to the spatial layout of individual ramets. This is the essence of the concept introduced by Lovett-Doust [2], assuming two expansion strategies—‘phalanx’ and ‘guerrilla’. The ‘phalanx’ strategy is represented by species that develop a slowly advancing front with a high density of ramets, allowing for continuous spreading within a community. On the other hand, species representing the ‘guerilla’ strategy infiltrate the surrounding vegetation with widely spaced ramets. According to Lovett-Doust [2], it is a ‘sampling’ of the environment allowing rapid growth, whereas the ‘phalanx’ strategy is a conservative process of consolidation and slower radial spread, causing exclusion of other plants from the clonal territory. 

The phalanx strategy, according to Schmid and Harper [3] and Sackville Hamilton et al. [4], generally increases the competitive strength of clonal species, whereas Lovett-Doust [2] and Humphrey and Pyke [5] suggest that it enables species to consolidate or maintain favorable patches within a community. The available literature unambiguously states that species representing the ‘guerrilla’ strategy have an advantage over ‘phalanx’-type species in exploring and conquering new areas and exploiting open space [5,6]. However, the ‘phalanx’-type species perform better on surfaces with a high density of other species [5]. 

Within recent decades, the rhizomatous grass *Calamagrostis epigejos* has been one of the most expansive clonal species, degrading various grassland communities in Europe [7,8,9,10,11] as well as in North America [12]. We understand the term ‘degradation’ of the plant community as a decline of species richness and evenness, caused by the strong expansion of *Calamagrostis epigejos* [13,14]. This species is one of the perennial clonal weeds with highly expansive features [7,15,16]. Due to strong competitive characteristics, high biomass productivity [11], and allelopathic properties [17,18], *C. epigejos* leads to a rapid decrease in the diversity of communities where it emerges. This problem is particularly relevant in the case of grassland ecosystems, where recent, rapid, and aggressive expansion of *C. epigejos* has been recorded [8,9,19]. *C. epigejos* produces two types of tillers: intra-vaginal and extra-vaginal [20]. The development of the first type leads to compact bunchgrass forms, whereas in the second case laterally spreading shoots form relatively long and loose stolons, creating the sodgrass-growth form [21].

Despite many studies published to date analyzing the role of various drivers affecting *Calamagrostis epigejos* spreading [7,15,22], the mechanism of its expansion still remains relatively unclear. There is also a lack of information about the influence of *C. epigejos* ramet density on the morphological characteristics of the species, which could be useful in predicting the future changes of plant communities affected by the colonization and growth of *C. epigejos*. Therefore, the objectives of this study were as follows: I. to determine the relationships between the biometric variability of *Calamagrostis epigejos*, its ramet density, and habitat properties; II. to determine the relationships between clone architecture, species diversity parameters, and the productivity of grassland communities; and III. to determine the expansion strategy of *Calamagrostis epigejos* within mesic meadows, representing three stages of their degradation.

## 2. Materials and Methods

### 2.1. Data Collection

The study was conducted in the Central Sudetes Mountains in an area encompassing hills of 400–550 m a.s.l. (NW: 50° 35.170 N, 16° 26.564 E; SE: 50° 33.320 N, 16° 30.420 E) in mesic meadows (*Arrhenatheretalia* type) degraded by the *Calamagrostis epigejos* expansion. Ten 10 m transects were set up within those meadows. Each transect crossed, perpendicularly, the front of a developed patch with *Calamagrostis epigejos*. Within each transect, sampling plots of 0.56 m^2^ (frame 75 × 75 cm) were set up at the distances of 1, 5, and 10 m from the first *C. epigejos* individuals, toward the interior of the degraded patches. This included areas with *C. epigejos* initial coverage (on average 49.5%), intermediate coverage (73.5% on average), and advanced coverage (90.7% on average). On each plot, the location of each individual ramet was projected using a frame (0.56 m^2^ area; 5 × 5 cm mesh diameter) based on the Pottier and Evette method [21]. The frames were then used for mapping the pattern of all ramets’ distribution within sampling plots, representing successive stages of *Calamagrostis epigejos* expansion. Ten individual ramets of *C. epigejos* were chosen for the biometrical measurements in each sampling plot. For these ramets, the following parameters were measured: ramet density (number/plot), ramet height (cm), culm mass (g/ramet), culm diameter (cm), leaf mass (g/ramet), leaf number (number/ramet), aboveground vegetative mass (overground part of the ramet without the panicle) (g/ramet), panicle length (cm), panicle mass (g/ramet), and runner mass (g/plot).

The vegetative mass was used to estimate the self-thinning of *Calamagrostis epigejos* along the transects.

Apart from the *Calamagrostis epigejos* biomass, random samples of co-occurring vegetation biomass were collected from all 30 sampling plots. For this purpose, another frame of 100 cm^2^ area was used. The five randomly chosen sampling points were set up within each study plot. Biomass of accompanying species was divided into monocotyledons and broad-leaved forbs within samples. In the laboratory, the samples were dried at 85 °C until dry mass was obtained. The entire field survey was carried out within 7 consecutive days to minimize the effect of time on collected data.

In order to investigate the changes, the expansion of the *Calamagrostis epigejos* causes in the properties of the habitat, soil samples were taken from each plot. The regular scheme of soil collection sites within each sampling plot was used: the pattern of dots like number 5 on a die. The material was gathered with a cylinder (10 cm diameter and 15 cm height). The surface layer of undecomposed organic matter was removed. Five individual samples of the same volume were then joined into a collective sample. In the laboratory, the samples were dried at room temperature and, as dried samples, sifted through a sieve with 2 mm mesh diameter. The samples were later studied for pH, organic matter content after burning 2 g of soil in a muffle furnace at 600 °C for 6 h, total nitrogen content (Kjeldahl method), and exchangeable phosphorus content determined colorimetrically after extraction in 0.5 M sodium bicarbonate (pH 8.5). Soluble forms of potassium, calcium, and magnesium were extracted with 1 M ammonium acetate (pH 7.0) [23,24]. The measurements were made using a Varian SpectrAA 200 spectrometer working in emission mode for calcium and potassium and in atomic-absorption mode for magnesium.

In order to calculate the value of the potential annual heat load on the basis of exposure and slope angles measured in the field, McCune’s equation [25] was used:

rlc = 0.339 + 0.808 × cos(L) × cos(S) − 0.196 × sin(L) × sin(S) − 0.486 × cos(A) × sin(S)

where rlc = potential annual heat load (MJ cm^−2^ year^−1^), A = exposure wrapped with regard to NS axis expressed in radians, S = slope angle in radians, and L = latitude.

### 2.2. Statistical Analysis

Statistical analyses were conducted using STATISTICA v. 13 [26] and RStudio (v. 2022.2.1.461) [27]. The consistency of data with a normal distribution was tested using the Shapiro–Wilk test. Data for which a normal distribution was obtained were analyzed with parametric methods: Pearson’s correlation coefficient and variance analysis with Tukey’s honest-significant-difference test. Data for which a normal distribution was not obtained were analyzed using non-parametric methods: Spearman’s rank-correlation coefficient and Kruskal–Wallis one-way analysis of variance. In order to determine the influence of habitat variables on selected biometric parameters of *Calamagrostis epigejos*, multiple regression analysis was used. Calculations were done for ln-transformed data and then standardized. Stepwise progressive analysis was used to build a model. In order to determine the relation among biometric properties of *C. epigejos*, principal component analysis (PCA) was used.

In order to determine the spatial distribution of individual *Calamagrostis epigejos* ramets, spatial autocorrelation analyses were performed using the software GeoDa v 1.20 [28]. The approach developed by Pottier and Evette [21] was used. Point data were transformed into lattice data by counting the number of tillers into each cell of the newly implemented grid. Moran’s I coefficient was calculated as the measure of the spatial autocorrelation for increasing spatial scales: 1 × 1 cm, 2.5 × 2.5 cm, 5 × 5 cm and 7.5 × 7.5 cm. According to Aubry [29] the coefficient was calculated for a spatial lag that integrated only the yi and yj pairs, as the observed values in cells i and j separated by a distance included in the lag boundaries. 

The species diversity was assessed for each sampling plot by the number of species, diversity index and evenness index. The Shannon–Wiener diversity index was calculated according to the formula H′ = −Σ (pi × ln pi), and the evenness index was calculated as J′ = H′/ln S, where pi = ni/N; ni = the abundance of the species expressed as its cover; N = the sum of abundances of all species, and S = the total number of species. The MVSP v. 3.131 package [30] was used for calculations of all diversity indices.

## 3. Results

### 3.1. Clonal Architecture of Calamagrostis epigejos

The conducted calculations did not reveal significant changes in the mean height of an individual ramet (H = 0.544; *p* = 0.7617), ramet culm mass (H = 2.261; *p* = 0.323), culm diameter (F = 1.057; *p* = 0.361), leaf mass (H = 1.492; *p* = 0.474),number of leaves (H = 1.163; *p* = 0.5591), vegetative mass (H =3.480; *p* = 0.1755), panicle length (F = 0.6078; *p* = 0.552), panicle mass (F = 0.0326; *p* = 0.968), or runner mass (H = 4.523; *p* = 0.1038) among different stages of *Calamagrostis epigejos* expansion (Table 1).

**Figure 1:**
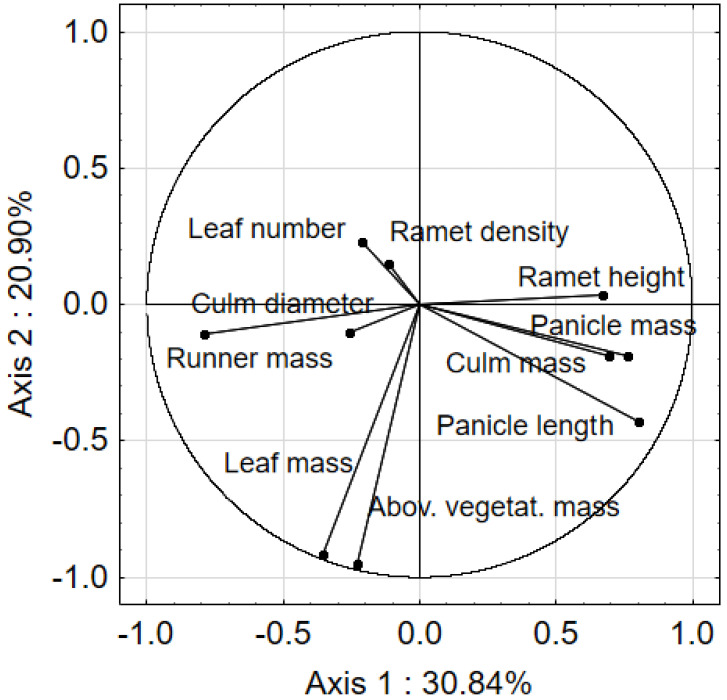
Ordination of biometric parameters of *Calamagrostis epigejos* in the space of two PCA axes.

In order to determine the relation among biometric properties of *Calamagrostis epigejos*, PCA was used. The main variation range was determined by the two first canonical axes, for which eigenvalues were recorded at 3.08 and 2.09. The cumulated variance percentage for the first and the second axis was, respectively, 30.84 and 51.74% (Figure 1). The first PCA axis was strongly correlated with panicle length (r = 0.801), runner mass (r = −0.792), panicle mass (r = 0.763), ramet culm mass (r = 0.693), and ramet height (r = 0.670). The second PCA axis showed a strong correlation with the vegetative mass (r = −0.951) and leaf mass (r = −0.913) of *C. epigejos*. It should be emphasized that as the density and coverage of *C. epigejos* increased, the results of the main-component analysis showed decreased culm mass and individual ramet height. 

No symptoms of self-thinning were observed. The vegetative mass of ramets did not differ significantly between the plots within transects, despite the significantly increasing density of the tillers.

### 3.2. Relation between Clone Architecture and Species Diversity in Various Spatial Scales

The *p* values indicate significant correlations between the values of Moran’s I coefficient for 1 cm (Rs = 0.452; *p* = 0.012) and 7.5 cm in the spatial scale and the total number of species (r = −0.4458; *p* = 0.014). In the case of the Shannon–Wiener index, significant correlations were recorded only for the 1 cm spatial scale (Rs = −0.591; *p* = 0.0006). No significant correlations were found for other variables.

The results of multiple regression analysis conducted for all measured biometric parameters of *Calamagrostis epigejos* and the total number of co-occurring species showed a significant influence of *C. epigejos* ramet density and ramet culm mass on the diversity of grassland communities (Table 2).

### 3.3. Relationship between Environmental Variables and Clonal Architecture

In order to determine the relationships between the studied biometric features of *Calamagrostis epigejos* and habitat properties, multiple regression analysis was used. The results are shown in Table 3. The final analysis of residuals performed showed their normal distribution (Shapiro–Wilk test), their lack of autocorrelation (Durbin–Watson test), and the linear character of the model.

Multiple regression results indicated soil depth, necromass deposed on the soil surface, biomass of the accompanying species, pH, and heat load as the habitat factors that most frequently and strongly affected the studied morphological features of the *Calamagrostis epigejos* individuals. Some other variables, including nutrients, also influenced some of those factors but in an equivocal way. 

### 3.4. Spatial Pattern of Calamagrostis epigejos Ramets

**Figure 2:**
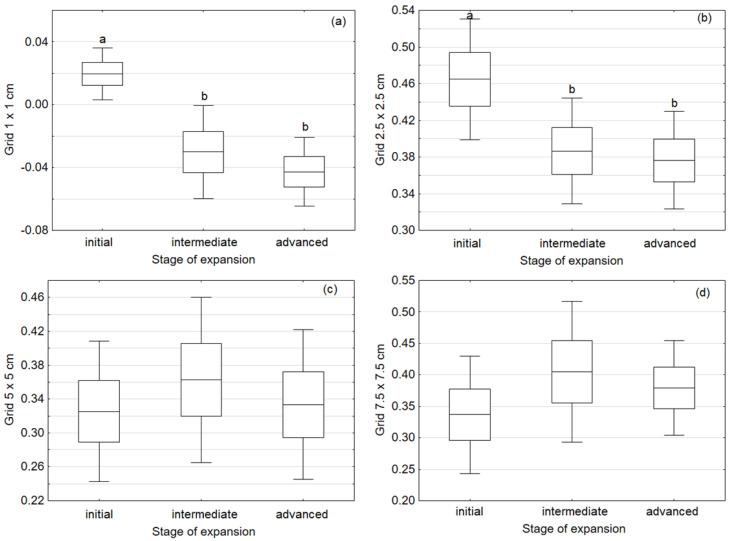
Mean values of Moran’s I coefficient for four resolution scales: 1 × 1 cm (**a**), 2.5 × 2.5 cm (**b**), 5 × 5 cm (**c**), and 7.5 × 7.5 cm (**d**). Means with standard error values (boxes) and confidence intervals (whiskers) for plots are given. Different letters indicate significant differences obtained after Tukey’s test or the Kruskal–Wallis test (*p* ≤ 0.05).

Significant differences in *Calamagrostis epigejos* ramet aggregation were recorded in the two following spatial scales: 1 cm (H = 11.56; *p* = 0.003) and 2.5 cm (F = 3.41; *p* = 0.04). The spatial structure of ramet distribution changed along the expansion gradient of *Calamagrostis epigejos*—from clusters in initial stages to random or regular in advanced stages, which was indicated by Moran’s indices for the 1 × 1 and 2.5 × 2.5 cm grids. No significant differences in mean Moran indicators for grids of 5 × 5 cm (F = 0.246; *p* = 0.783) or 7.5 × 7.5 cm (F = 0.682; *p* = 0.514) were found in the spatial scale (Figure 2). 

The analysis of Moran’s indices did not give unequivocal results. For the finest grid, 1 × 1 cm, at all distances the values were close to zero, indicating a clustered pattern of ramet distribution. At all other resolution levels, we found values above 0.3, which indicates positive spatial autocorrelation and a random or regular distribution pattern. 

Considering the correlations between Moran’s indicator and the habitat parameters, there was a significant correlation between Moran’s indicator values for the 2.5 cm scale and the exchangeable forms of potassium (Rs = 0.385; *p* = 0.03) and calcium (Rs = 0.49; *p* = 0.006) and the heat load (Rs = −0.441; *p* = 0.015).

## 4. Discussion

### 4.1. Clonal Architecture of Calamagrostis epigejos

A significant increase in *Calamagrostis epigejos* coverage and ramet density was noted along the degradation gradients of the studied meadows. However, it is noteworthy that along with the increase in *Calamagrostis epigejos* density, no significant variability of its biometric parameters was observed. The lack of influence of increased ramet density on the biometric parameters of *C. epigejos* can be explained by the species biology. Due to its ability to sustain connections between individual ramets of the clonal species and the translocation of nutrients between particular modules [31], the clonal species probably can adapt to variability in habitat conditions. The functioning of individual ramets as one clone allows the species to limit the phenomenon of intra-species competition for resources, which, in turn, causes efficient competition with other species. Furthermore, different environmental factors may influence the growth and competitive abilities of grassland species even in the same place. Price et al. [32], studying grassland-assembly patterns, stated that biotic-assembly rules were of crucial importance aboveground, while belowground assembly is driven by abiotic factors. 

### 4.2. Influence of Environmental Factors on Clonal Architecture

The results showed significant relationships among some of the physico-chemical properties of soil on the *Calamagrostis epigejos* biometric variability. The analyses showed a negative correlation between soil pH and the density of *C. epigejos* ramets, individual ramet height, and runner mass. It can be assumed that the increase in soil pH leads to limited macro-element assimilation, mainly magnesium and nitrates [33], which results in the impeded growth and development of *C. epigejos*. Such a phenomenon was also observed in the case of forest and forest-clearing plant communities [16]. According to Rebele [34] and Lammerts et al. [35], concentrations of available phosphorus and potassium stimulated the growth of *C. epigejos*. However, our research indicated only a stimulating influence of potassium on the growth of runner mass, with a negative impact on the mass of the panicles and the culm. In the case of phosphorus concentrations, the results demonstrated a negative correlation with runner mass. Lehmann and Rebele [36] and Tůma et al. [37] reported that growth of *C. epigejos* biomass was stimulated under the influence of increased nitrogen availability, which was also confirmed by our results. 

Plant necromass deposited on the soil surface appeared to be one of the factors negatively influencing ramet height and its culm mass, while positively influencing panicle mass at the same time. The necromass is formed mainly by dead *Calamagrostis epigejos* ramets. According to Holub et al. [38] and Pruchniewicz and Żołnierz [11], production of a thick layer of slowly decomposing necromass is one of the basic causes of the competitive advantages of *C. epigejos*. Such a thick necromass layer inhibits germination of both seeds deposited in the soil seed bank and those coming from the seed rain and remained isolated from the mineral ground [11]. In this study, we also observed inhibition of ramet growth. Apart from the physico-chemical properties of soil, our study showed a significant influence of heat load and soil depth on the *Calamagrostis epigejos* biometric variability and growth. On plots characterized by a high heat load, the species showed an increase in leaf number, though the panicle length and mass decreased at the same time. The negative impact of soil thickness on runner mass is consistent with our previous study [16] and is related to the increase in root density.

### 4.3. Spatial Pattern of Calamagrostis epigejos Ramets and Its Influence on Species Diversity

We found significant differences in the level of ramet aggregation, with regard to the two smallest scales: 1 × 1 cm and 2.5 × 2.5 cm.

*Calamagrostis epigejos* in our study indicated different characteristics of Moran’s I changes, within the range of the four resolution levels, as compared to values concerning this species obtained in the survey of Potter and Evette [21]. They also observed an increase in the aggregation along the growing spatial grid. However, in our study, that process was much faster and reached almost twice as high values at the levels of 2.5 cm and higher.

We established the starting points of our transects where the domination of *Calamagrostis epigejos* became distinctly visible. The tiller density achieved there was less than 40% of the maximum in the last stage of expansion in our study, which is similar to the value (ca. 600 tillers/m^2^) reported by Potter and Evette [21]. The space autocorrelation analysis indicated high tiller aggregation, typical for the phalanx strategy already in the first plots of the transects. Thus, we rejected our hypothesis that within the space of our transects the transition between the guerilla and phalanx strategies occurs. The guerilla form is probably restricted to the very early ‘outposts’, where the expansion of *C. epigejos* starts. This strategy enables the species to exploit and conquer new surfaces, as well as to escape from places of low resources and high competitive stress to more favorable microsites [2,6]. According to Humphrey and Pyke [5], it can be generalized that guerrilla clonal plants are advantageous over phalanx ones in exploiting open spaces, which is common for early-succession stages. Żołnierz [8] observed such a phenomenon in disturbed sites within patches of dry grasslands, e.g., around pits dug by animals. In our study, *Calamagrostis epigejos* apparently represented the ‘phalanx’ strategy within degraded habitats. Due to that, the species is able to dominate mesic meadows in a short time and, therefore, the affected meadows quickly lose their high species diversity. In fact, we found no visible gaps within the vegetation cover in the studied meadow patches, so it can be assumed that they represent later stages of succession, where there is phalanx growth.

## 5. Conclusions

Only the phalanx strategy was observed within the studied range of the *Calamagrostis epigejos* expansion in the mesic mountain meadows. Probably, the guerilla strategy is restricted there only to initial stages of the species expansion. We noted significant influences of some environmental factors on the variability of *Calamagrostis epigejos* biometric features. The study did not reveal any relationship between increasing density of *Calamagrostis epigejos* and the phenomenon of its ramet self-thinning.

## Figures and Tables

**Table 1:** Mean values of the biometric parameters *of Calamagrostis epigejos* (±SE) in successive stages of its expansion. Different letters in each column (a, b) show significant differences obtained after the Kruskal–Wallis test (*p* ≤ 0.05).

	Stage of Expansion:		
	Initial (1 m)	Intermediate (5 m)	Advanced (10 m)
Ramet density	219.20 ± 32.62 a	509.00 ± 53.46 b	552.90 ± 33.12 b
Ramet height	89.61 ± 6.82	92.28 ± 6.47	95.86 ± 6.82
Culm mass	1.14 ± 0.05	1.01 ± 0.06	1.16 ± 0.09
Culm diameter	2.31 ± 0.18	2.60 ± 0.14	2.36 ± 0.13
Leaf mass	0.73 ± 0.49	0.23 ± 0.03	0.69 ± 0.46
Leaf number	2.07 ± 0.08	2.20 ± 0.10	2.17 ± 0.15
Aboveground vegetative mass	1.87 ± 0.48	1.24 ± 0.07	1.84 ± 0.44
Panicle length	16.90 ± 1.47	15.13 ± 1.22	16.93 ± 1.26
Panicle mass	0.37 ± 0.05	0.35 ± 0.04	0.35 ± 0.05
Runner mass	347.78 ± 24.14	462.72 ± 69.83	437.12 ± 56.54

**Table 2:** Results of multiple regression analysis (stepwise progressive method) of *Calamagrostis epigejos* biometric parameters and the total number of species. The analyses were conducted for standardized and ln variables.

	BETA	r	R^2^	F	df	*p*
Total Number of Species (s)						
Ramet density	−0.542	−0.600	0.020	5.949	4.25	0.0017
Ramet culm mass	−0.532	−0.535	0.274			

**Table 3:** Multiple regression results for biometric parameters of *Calamagrostis epigejos* and habitat factors. The analyses were conducted for ln and transformed variables.

	BETA	r	R^2^	F	df	*p*
*Calamagrostis epigejos* coverage						
Graminoid biomass	−0.632	−0.632	0.399	18.630	1.280	<0.001
Ramet density						
Graminoid biomass	−0.463	−0.531	0.514	5.087	5.240	0.002
pH	−0.667	−0.576				
Ramet height						
Ca	0.282	0.412	0.822	17.770	6.230	<0.001
Necromass	−0.823	−0.730				
Soil depth	0.879	0.670				
pH	−0.407	−0.466				
Ramet culm mass						
Necromass	−0.709	−0.591	0.451	7.120	3.260	0.001
Soil depth	1.315	0.621				
K	−0.795	−0.467				
Culm diameter						
Non-significant (ns)	ns	ns	0.474	7.804	3.260	<0.001
Leaf mass						
Soil depth	−0.982	−0.399	0.329	3.075	4.250	0.034
Leaf number						
N	0.623	0.572	0.404	5.873	3.260	0.003
Forbs biomass	0.444	0.447				
Heat load	0.349	0.409				
Vegetative mass						
Non-significant (ns)	ns	ns	0.225	2.524	3.260	0.080
Panicle length						
Heat load	−1.402	−0.674	0.779	16.911	5.240	<0.001
pH	0.287	0.394				
Panicle mass						
Heat-load	−3.164	−0.719	0.789	6.130	11.180	<0.001
Necromass	2.197	0.697				
K	−0.938	−0.630				
Forbs biomass	0.524	0.555				
N	0.471	0.551				
Runner mass						
pH	−0.673	−0.770	0.863	19.783	7.220	<0.001
Heat load	0.477	0.657				
Total graminoids and forbs biomass	−0.312	−0.612				
Soil depth	−1.256	−0.770				
K	0.722	0.649				
*p*	−0.564	−0.549				

## Data Availability

Not applicable.
